# Nitrate Leakage from Deciduous Forest Soils into Streams on Kureha Hill, Japan

**DOI:** 10.1100/tsw.2001.360

**Published:** 2001-11-21

**Authors:** H. Honoki, T. Kawakami, H. Yasuda, I. Maehara

**Affiliations:** Toyama Science Museum, Toyama-city, Japan

**Keywords:** nitrogen saturation, net nitrification, net nitrogen mineralization, C/N ratio, Japan, deciduous forest

## Abstract

Nitrate leakage from deciduous forest soils into streams was investigated for two adjacent hills. Many of the streams on Kureha Hill, located in Toyama City, Japan, have extremely high nitrate concentrations. The nitrate concentration of Hyakumakidani, one of the streams on Kureha Hill, averaged 158 μeq l and reached 470 μeq l during an episodic event. In contrast, the streams on Imizu Hill, adjacent to Kureha Hill, had low concentrations, below 15 μeq l. Even during an episode, the nitrate concentrations increased to no more than 75 μeq l.Both areas have similar blown forest soils, C/N ratios in O horizons, and vegetation consisting primarily of deciduous trees. However, soil incubation experiments, which lasted for 4 weeks, revealed that the nitrification rates in the surface soils of Kureha Hill were much higher than in the soils of Imizu Hill.

### INTRODUCTION

One of the current environmental concerns is nitrogen saturation under which nitrogen is no longer the limiting factor of the forest ecosystem's growth and nitrate is leached into the soil solution and stream water by the forest ecosystem. Nitrate leaching can adversely affect soil acidification, leading to cation losses and aluminum leaching from soils, which are harmful to the forest and aquatic ecosystems[1,2]. The hypotheses regarding the progresses of nitrogen saturation are well-documented[1,3,4] and many symptoms of nitrogen saturation have been reported in Europe[5,6,7], the U.S.[8,9], and Japan[10,11]. However, the factors that cause nitrogen leaching from forested watersheds remain unknown. In addition to excess atmospheric nitrogen loading, which appears to be one of the causes for nitrate leaching[8,12], C/N ratios of the forest floor have been considered an important factor. A C/N ratio of 25 has been suggested as a threshold for the leaching of nitrate into soil waters of European forests[13]. Dise et al.[14] found that C/N ratios, in addition to nitrogen loading, are related to nitrate leaching in runoff and seepage water from forests across Europe. Mitchell et al.[15], who examined Japanese forested watersheds, hypothesized that the high nitrogen mineralization and nitrification rate in Japanese forests caused a high rate of nitrate leaching throughout the year. Tietema et al.[16] introduced an "N status" involving 15 variables to explain the nitrate leaching based on the results of NITREX experiments.

* Corresponsing author. Tel: +81-76-491-2123
E-mails: honoki@tsm.toyama.toyama.jp; Kawakami, T.: kawakami@pu-toyama.ac.jp; Yasuda, H.: yasuda@fes.pref.toyama.jp; Maehara, I.: issei-m@p1.tcnet.ne.jp
© 2001 with author.

In the current study, we investigated two adjacent hills in Japan with different nitrogen statuses. The watersheds on Kureha Hill are considered to be suffering from nitrogen saturation[11], while the watersheds on Imizu Hill, adjacent to Kureha Hill, are considered to be under nitrogen-limiting conditions. Many of the streams on Kureha Hill contain a high concentration of nitrate throughout the year, while the streams on Imizu Hill contain less than 10 μeq $l^{-1}$ nitrate except during extremely high-discharge periods caused by heavy rainstorms.

The aim of this paper is to evaluate the factors that regulate nitrate leaching from Japanese forested watersheds to stream water.

### SITE DESCRIPTION

Kureha Hill and Imizu Hill are located near Toyama City (36°41′N, 137°9′E), Japan and adjacent to each other. The locations of the hills are shown on the topographic map (Fig. 1). Kureha Hill overlays alternating layers of sand and mud deposited by Quaternary Lake; Imizu Hill overlays alternating sand and mud layers from Neogene. The soils of both hills are classified as brown forest soils[17](Fig. 2). Both hills are 80% covered by deciduous trees, primarily 40-year-old Konara (*Quercus serrata*). The rest of Kureha Hill is covered by bamboo (*Phyllostachys pubescens*) and afforested 50-year-old coniferous Sugi (*Cryptomeria japonica*), while the rest of Imizu Hill is covered by 50-year-old coniferous Sugi. There are many small streams with watershed areas of less than 10 ha on both hills. The stream-water qualities on both hills are characterized by low pH, low acid neutralizing capacity (ANC), and low concentrations of base cations; however, the nitrate concentrations are quite different. Most of the streams on Kureha Hill have high concentrations of nitrate throughout the year, while the streams on Imizu Hill have low concentrations of nitrate. Hyakumakidani, with a 4-ha watershed, is one of the streams on Kureha Hill. Sannokuma, with a 1.9-ha watershed, is on Imizu Hill. The two watersheds are only 5 km apart from each other. No agricultural areas exist within the watersheds.

### METHOD

#### Precipitation and Stream-Water Analyses

Precipitation was measured by a rain gauge and bulk precipitation was collected weekly in a 20-cm-diameter funnel collector placed in an open area within the Hyakumakidani watershed. Meanwhile, throughfall was collected weekly in an 18-cm-diameter funnel collector placed under a Konara canopy within the Hyakumakidani watershed. Stream water was sampled from Hyakumakidani and Sannokuma weekly or biweekly. In addition, both stream waters were sampled irregularly during heavy rainfall in order to investigate the acid-base chemistries at increased discharge. Discharges of both streams were recorded every 10 min at weirs placed in the streams.

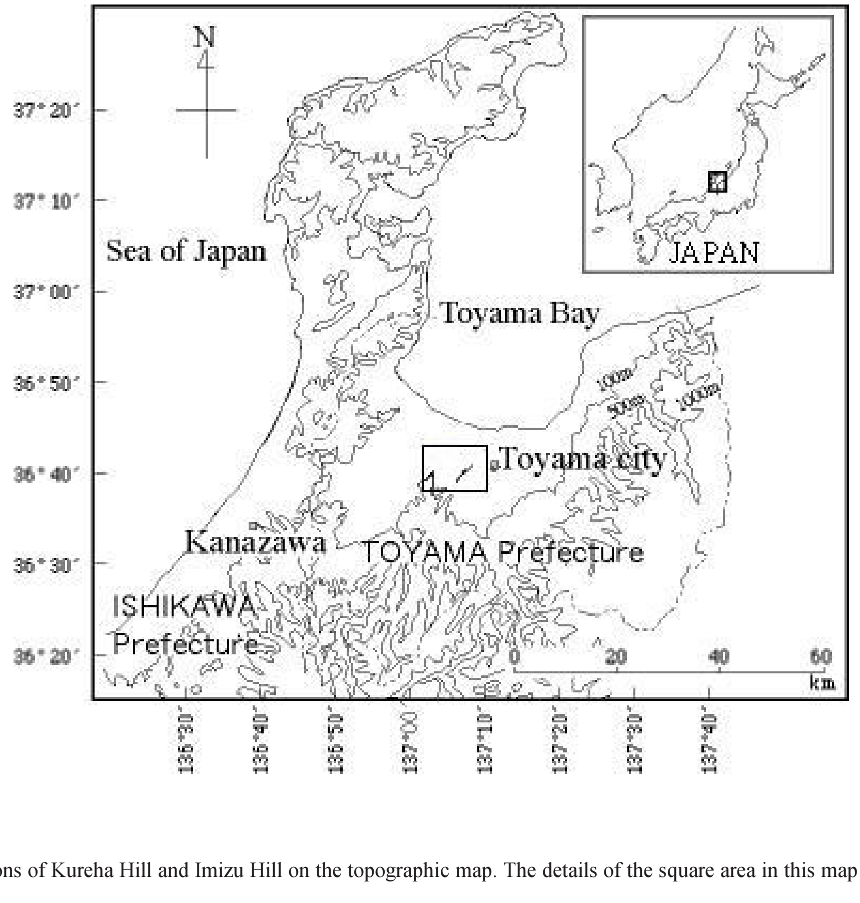

**FIGURE 1**. The locations of Kureha Hill and Imizu Hill on the topographic map. The details of the square area in this map are illustrated in Fig. 2.

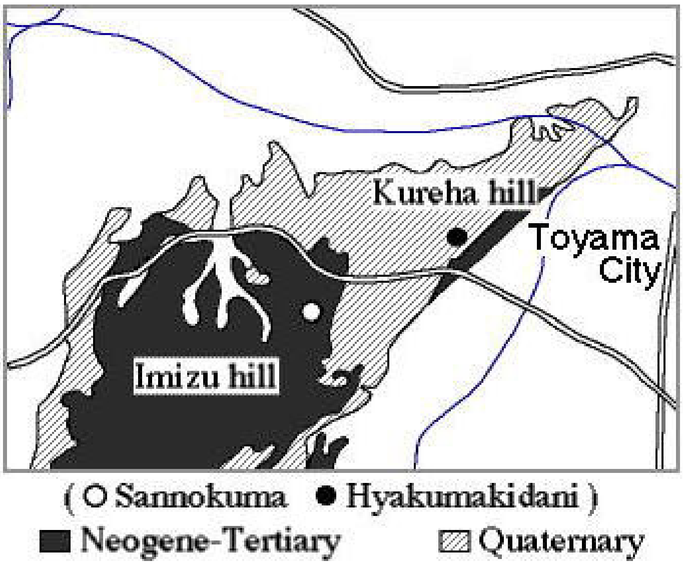

**FIGURE 2**. The locations of Hyakumakidani on Kureha Hill and Sannokuma on Imizu Hill are superimposed on the geological map.

On June 16, 2001, a synoptic survey was conducted to provide water chemistries of the streams on both Kureha Hill and Imizu Hill. In the survey, three streams in addition to Hyakumakidani on Kureha Hill were sampled and nine streams in addition to Sannokuma on Imizu Hill were sampled . All stream- and rainwater samples were filtered over 0.45-µm filters in the laboratory within 1 h after sampling. ANC was measured by Gran titration. Dissolved components were analyzed by ion chromatography. Dissolved total nitrogen (DTN) was determined by an ultraviolet absorption method after degradation by potassium peroxodisulfate at 120°C.

### Nitrogen Budget Calculations

Nitrogen budgets were calculated for the Hyakumakidani and Sannokuma watersheds. The amount of precipitation and the chemical components of bulk precipitation obtained within the Hyakumakidani watershed were used for both watersheds in calculating the nitrogen budget. The nitrogen inputs were obtained by multiplying the amount of precipitation by ammonium-plus-nitrate concentrations in the bulk precipitation. We used bulk precipitation instead of throughfall because throughfall could have larger space variability and cause difficulty in evaluating the internal nitrogen cycle, especially for deciduous trees. The nitrogen inputs calculated by using throughfall and DTN were used as references. The nitrogen losses at a specific time from a watershed can be calculated by multiplying nitrate concentration of the stream by an amount of discharge at the same time. However, the nitrate concentrations were so much fluctuated with the discharge rates of the Hyakumakidani stream and of the Sannokuma stream that we estimated the nitrate concentrations from the discharge after establishing relational equations between nitrate concentrations and the discharge rates. The nitrogen losses were calculated every hour by multiplying the estimated nitrate concentrations by the amount of measured discharge. The ammonium and organic nitrogen were not considered in calculating the nitrogen losses since the ammonium concentrations were negligible compared to nitrate concentrations and DTN concentrations were as same as nitrate concentrations for the both streams.

### Net Nitrogen Mineralization and Net Nitrification

Soil samples were taken from 5-, 10-, and 30-cm depths of three plots within the Hyakumakidani and Sannokuma watersheds. Samples from the same depth of each watershed were mixed to produce composite samples for incubation. Each composite sample was divided into three parts. Two parts were for the incubation and the other was for the measurement of initial concentrations. Incubation continued for 4 weeks at 15°C with the moisture content at 60% of the water-holding capacity. After incubation, ammonium and nitrate ions were extracted from the soils using 0.2 mol $l^{-1}$ KCl. Ammonium was analyzed colorimetrically by the phenol-hypochlorite method, and nitrate was analyzed by ion chromatography. The net nitrogen mineralization was calculated as the difference between the sum of nitrate and ammonium concentrations before and after incubation. The net nitrification was calculated as the difference between the nitrate concentrations before and after incubation.

## RESULTS AND DISCUSSION

### Acid-Base Chemistry of the Streams

The water qualities of the streams on the two hills are shown in Table 1, together with the average concentration of bulk precipitations. The data for bulk precipitation and Hyakumakidani are shown in an averaged value of data collected from September 1998 to August 2001. The data for Sannokuma are averaged value of data collected from October 2000 to August 2001. The other

**TABLE 1**
**Chemical Characteristics of Bulk Precipitation and Streams on Kureha Hill and Imizu Hill**

| | Area | pH | ANC<br>μeq $l^{-1}$ | $C_B$<br>μeq $l^{-1}$ | $SO_4^{2-}$<br>μeq $l^{-1}$ | $NH_4^+ + NO_3^-$<br>μeq $l^{-1}$ |
|---|---|---|---|---|---|---|
| Precipitation | Kureha | 4.9 | | 133 | 51 | 53 |
| Hyakumakidani | Kureha | 5.2 | -4 | 540 | 155 | 160 |
| Kureha Hill average (n=3) | Kureha | 6.0 | 70 | 584 | 109 | 148 |
| Sannokuma | Imizu | 5.4 | 16 | 377 | 87 | 9 |
| Imizu Hill average (n=7) | Imizu | 6.6 | 54 | 413 | 120 | 8 |

$C_B$ denotes base cations, i.e., $Na^+ + K^+ + Mg^{2+} + Ca^{2+}$.

stream samples were collected on June 16, 2001. All the stream water had low ANC and low base-cation concentrations. Sulfate concentrations higher than those in bulk precipitations, which are sometimes observed in this area, indicated the existence of an internal source of sulfate, likely the sulfate originating from the oxidation of pyrite[18]. The acidity yielded by the internal sulfate might have leached the cations of the soils and resulted in low concentrations of cations, low pH, and low ANC in the stream water.

Nitrate concentrations were much different among the streams on the two hills. Most of the streams on Kureha Hill had over 90 μeq $l^{-1}$ of nitrate concentrations even in the growing season, while the streams on Imizu Hill had less than 15 μeq $l^{-1}$. The nitrate concentrations increased during high-discharge events for several streams on Kureha Hill. The changes in nitrate concentrations and ANC according to discharge for Hyakumakidani are illustrated in Fig. 3. Hyakumakidani has not only suffered from chronic nitrate-induced acidification but also from episodic acidification. A significant increase in nitrate concentrations and a decrease in ANC were observed coincidentally with an extremely high discharge. The highest nitrate concentration, which surprisingly increased to 470 μeq $l^{-1}$, was observed on September 12, 2000, when a rainstorm brought 196 mm of precipitation within 3 days after a 7-week drought. In general, the nitrate concentration increased with a heavy rainstorm after a drought in Hyakumakidani. These results support the hypothesis that drought followed by rewetting causes an initial flush of accumulated nitrate in the soils[19]. In Sannokuma, the nitrate concentrations increased to no more than 75 μeq $l^{-1}$ even during the episode on September 12, 2000.

Ammonium concentrations were less than 2 μeq $l^{-1}$ for all the streams. DTN concentrations were on the same level as nitrate concentrations, indicating that all the nitrogen components were oxidized into nitrate before leaching to the streams.

In Hyakumakidani, linear relationships between the nitrate concentrations and discharge were found when the discharge was expressed on a logarithmic scale as illustrated in Fig. 4, indicating that the nitrate concentrations depended only on stream discharge, regardless of the season. In Sannokuma, nitrate concentrations remained fairly low both in the dormant season and the growing season. Linear relationships were found only when the discharge rate exceeded 0.7 l $s^{-1}$ as illustrated in Fig. 5. Stoddard[1] hypothesized that no seasonality of nitrate concentrations was one of the evidences of a high stage of nitrogen saturation. However, Ohrui[20] argued that a constant nitrate concentration without seasonality was not a useful index of nitrogen saturation for Japanese forest, since the phenomenon could be attributed to the hydrological patterns particular to Japanese streams as well as high nitrogen mineralization and nitrification rate during the growing season caused by high temperatures and moisture. In Hyakumakidani, the hydrological patterns, which are illustrated in Fig. 3, are more similar to those of North America and Europe than Ohrui's sites because we receive much snow in winter in this area. Furthermore, we found the absence of seasonality as indicated in Fig. 4 even after eliminating the influence of the hydrological patterns. We think that Stoddard's hypothesis can be applied to the Hyakumakidani watershed and this watershed was in the high stage of nitrogen saturation. In this watershed, the soil surface is covered by snow from January to March, and the snowpack depth reaches at a maximum of 1 m. The higher nitrate concentration accompanied by higher discharge in winter suggested that nitrifiers in soils still worked effectively under the snowpack. Further study is needed to verify the activity of nitrifiers under low temperature in relation to nitrogen saturation.

### Nitrogen Budget

The nitrogen budgets are shown in Table 2 for Hyakumakidani and Sannokuma. In order to estimate the nitrogen concentrations, we used the relational equations between nitrogen concentrations and discharge rates shown in Figs. 4 and 5. We used the different equations before and after August 1999 for Hakumakidani, because the relationships between nitrogen concentrations and discharge rates have been changed after a drought occurred in August 1999. The nitrate concentrations after the drought increased about 1.5 times higher than those before the drought. The reason of this change is unknown, however, one of the possibilities is the change in the microbial activity for nitrification as far as our flow-path-simulation model using two-tank model indicated that the flow paths in the soil have not changed before and after the drought.

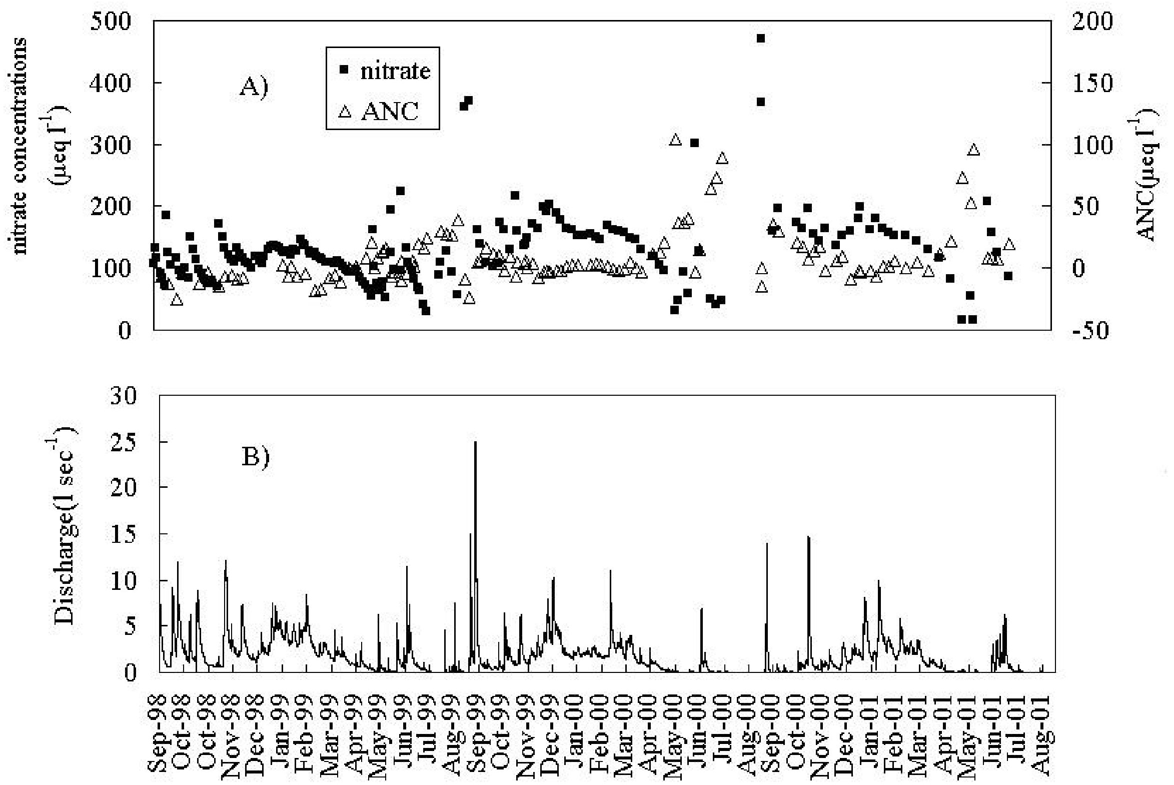

**FIGURE 3**. Time series of measured nitrate concentrations and ANC (A), and discharge (B) for Hyakumakidani.

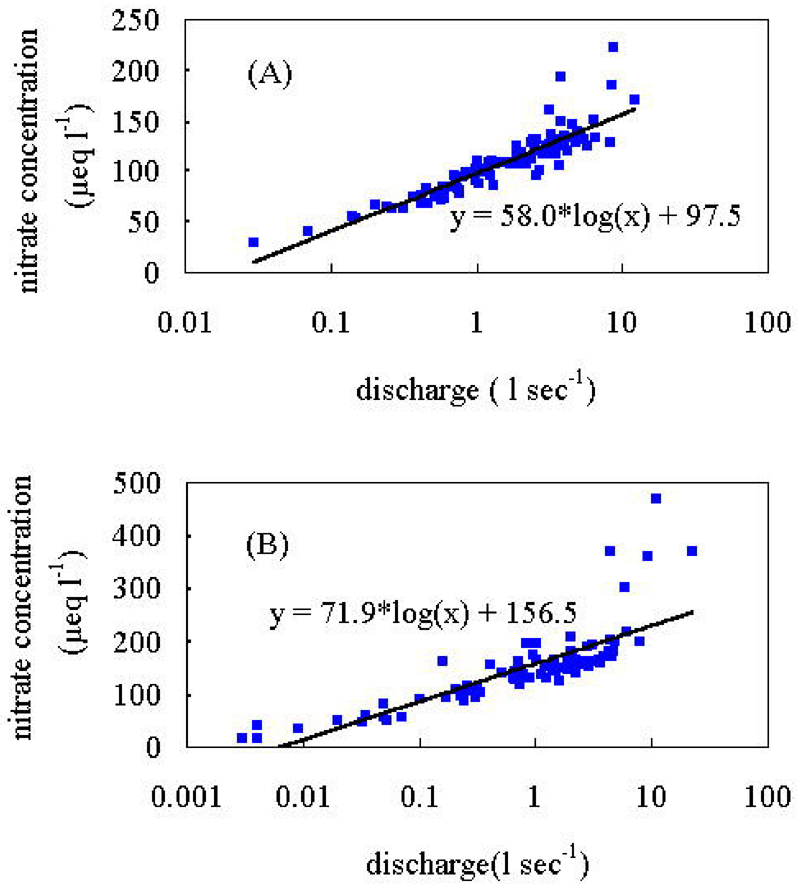

**FIGURE 4**. The relationship between nitrate concentrations and discharge for Hyakumakidani: (A) September 1998 to August 1999, (B) September1999 to August 2001.

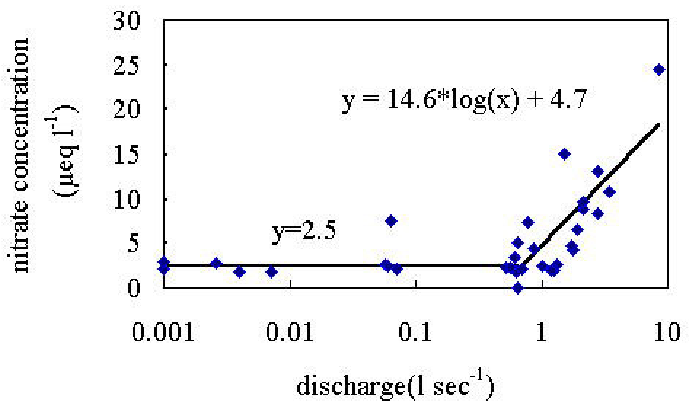

**FIGURE 5**. The relationship between nitrate concentrations and discharge for Sannokuma (October 2000 to August 2001).

**TABLE 2**
**Nitrogen Budgets of the Hyakumakidani and Sannokuma Watersheds**

| | Hyakumakidani | | | Sannokuma |
|---|---|---|---|---|
| Water Year | '98 Sep.- '99 Aug. | '99 Sep.- '00 Aug. | '00 Sep.- '01 Aug. | '00 Oct.- '01 Aug. |
| Precipitation (mm) | 2320 | 1821 | 2174 | 1823 |
| Nitrogen Input (eq $ha^{-1}$) | 1228 | 1028 | 1121 | 1038 |
| Nitrogen Output (eq $ha^{-1}$) | 2117 | 2277 | 1674 | 100 |

Hyakumakidani had a higher output of nitrogen than input, while for Sannnokuma, the output of nitrogen was much less than the input. When we used DTN in calculating the input instead of ammonium-plus-nitrate, the nitrogen input increased 18% on average. When we used throughfall instead of bulk precipitation, the DTN deposition increased 13%. Taking these factors into consideration, the loss of nitrogen exceeded the nitrogen input. These results implied that Hyakumakidani was in Stage 3 of nitrogen saturation according to Stoddard's definition[1] because the watershed became a net source of nitrogen. On the other hand, Sannokuma seemed to remain in a nitrogen-limiting condition. Nitrate leaching into streams has been reported in many watersheds in which the nitrogen loadings exceeded the threshold level. For examples, Dise et al.[21] found that the threshold level was around 10 kg N $ha^{-1}$ $year^{-1}$ from NITREX sites throughout Europe, while Peterjohn et al.[8] reported that U.S. forests receiving more than 4 kg N $ha^{-1}$ $year^{-1}$ of inorganic nitrogen exhibited variable stream-water losses. Mitchell et al.[15] found a significant relationship between nitrogen input and stream-water losses in Japanese forests. The threshold level was not clear from their data, however, nitrogen loading exceeding 10 kg N $ha^{-1}$ $year^{-1}$ seemed to cause significant losses of nitrogen to the streams. Most of the watersheds in these studies, except a few in Japan, had less output than input, indicating that some nitrogen had been retained in the watershed, even though the watersheds leached some nitrate into the streams. The input of nitrogen, 17.2, 14.4, and 15.7 kg N $ha^{-1}$ $year^{-1}$ for the 1998, 1999, and 2000 water years, respectively, exceeded these thresholds. It is possible for Hyakumakidani to attribute the exceeding loss of nitrogen to the elevated nitrogen loadings, however, in Sannokuma, severe nitrate leaching has not occurred even though the nitrogen input was likely to be the same as that of Hyakumakidani.

### Soils

The pH, base saturation, and C/N ratios for the O, A, and B layers of the forested watersheds for Hyakumakidani and Sannokuma are shown in Table 3. Low soil pH values were observed in all the layers, and base saturations were extremely low for the A and B layers of both watersheds. This is partly because of the sulfate yielded from an internal source; however, in addition to the sulfate, it is apparent that nitrate leached from the soils would have adversely affected soil acidification in Hyakumakidani. No significant differences in C/N ratios between Hyakumakidani and Sannokuma watersheds were found. The C/N ratios of the O layer, 15 and 18 for Hyakumakidani and Sannokuma, respectively, were much lower than 25, which is considered the threshold of a nitrogen leaching in European forests[13]. If the threshold could be applied to Japanese forests, C/N ratios below 25 for both water-

**TABLE 3**
**Soil Characteristics of the Studied Areas**

| | Hyakumakidani | | | Sannokuma | | |
|---|---|---|---|---|---|---|
| Depth (cm) | 0-4 | 4-40 | 40-68 | 0-5 | 5-30 | 30-55 |
| Classification | O | A | B | O | A | B |
| pH (KCl) | 3.6 | 3.8 | 4.0 | 3.8 | 4.0 | 4.1 |
| B.S. | 19.4 | 1.4 | 6.4 | 10.9 | 6.3 | 6.9 |
| Carbon content (g-C 100g dry soil$^{-1}$) | 4.9 | 1.4 | 1.1 | 5.4 | 2.1 | 0.56 |
| Nitrogen content (g-N 100g dry soil$^{-1}$) | 0.32 | 0.11 | 0.090 | 0.30 | 0.12 | 0.042 |
| C/N | 15 | 13 | 12 | 18 | 18 | 13 |

B.S. denotes base saturation sheds suggested that Sannokuma watershed, currently estimated thought to be under nitrogen-limiting conditions, was likely to fall into nitrogen saturation.

### Net Nitrogen Mineralization and Net Nitrification

Net nitrogen mineralization and net nitrification rates are shown in Table 4. The net nitrogen mineralization rates were not much different between the two watersheds and were within 5 to 564 µmol 100 g dry soil$^{-1}$ 6 weeks$^{-1}$, as observed in other Japanese watersheds[22]. On the other hand, high proportions of nitrification rate to nitrogen mineralization rate were observed in the soils of Hyakumakidani. These high proportions were distinctive of Hyakumakidani, while most parts of the decomposed nitrogen from organic compounds in Sannokuma remained as a form of ammonium. High proportions of nitrification to nitrogen mineralization were also observed in Fernow Experimental Forest, which has been thought to be under nitrogen saturation[9]. Aber et al.[4] proposed a modified hypothesis in which they predicted the reduction in nitrogen mineralization in stage 3 of nitrogen saturation. Different from their initial hypothesis, which predicted increases in both nitrogen mineralization and nitrification[3], their modified hypothesis predicted high proportions of nitrification to nitrogen mineralization at a high stage of nitrogen saturation. The high proportions found in Hyakumakidani and Fernow Experimental shows good agreement with their hypothesis.

Because nitrate is much more mobile than ammonium, these results indicated that a high conversion rate of nitrogen to nitrate was one of the reasons the Hyakumakidani watershed leached nitrate into the stream. Furthermore, higher net nitrogen mineralization and net nitrification rates in the soil near the surface than in mineral soils supported the hypothesis that an increased lateral flow in the sub-surface soil causes high nitrate concentrations during high-discharge periods[11].

We have no conclusive evidence about the causes of the difference in net nitrification rates between Hyakumakidani and Sannokuma. Tietema et al.[16] investigated the factors that regulated the nitrate leaching from European forests and found that even small disturbances in ecosystems, including nitrogen loading, moisture, temperature, and so on, might have a large effect on nitrogen leaching as the sites became nitrogen saturated. This suggests that an imperceptible difference in the nitrogen input between Hyakumakidani and Sannokuma could cause a large effect on the species of nitrifiers adaptable to the nitrogen-rich environment and to the following acidic environment.

### CONCLUSIONS

Forested watersheds and streams on two adjacent hills with different nitrogen statuses were investigated in order to clarify the causes of nitrogen saturation. The high nitrate concentrations

**TABLE 4**
**Net Nitrogen Mineralization and Net Nitrification**

| | Hyakumakidani | | | Sannokuma | | |
|---|---|---|---|---|---|---|
| Depth (cm) | 0-5 | 5-15 | 15-30 | 0-5 | 5-15 | 15-30 |
| Net nitrogen mineralization (µmol 100g$^{-1}$ dry soil 4weeks$^{-1}$) | 143 | 31 | 23 | 75 | 20 | 6 |
| Net nitrification (µmol 100g$^{-1}$ dry soil 4weeks$^{-1}$) | 150 | 32 | 23 | 11 | 2 | 1 |
| Net nitrification / Net mineralization | 1.05 | 1.03 | 1.00 | 0.15 | 0.1 | 0.17 |

observed in stream water on Kureha Hill indicated that the watersheds suffered from nitrogen saturation, while low nitrate concentrations indicated that watersheds on Imizu Hill, adjacent to Kureha Hill, remained under nitrogen-limiting conditions. Among the watersheds on Kureha Hill, Hyakumakidani was regarded as having a high stage of nitrogen saturation because nitrogen output exceeded nitrogen input twofold.

In order to evaluate the factors that regulate nitrate leaching from forested watersheds to stream water, the C/N ratios, net nitrogen mineralization rates and net nitrification rates were measured for Hyakumakidani watershed and Sannokuma watershed on Imizu Hill. The only difference between two watersheds was the proportion of net nitrification rate to net nitrogen mineralization rate. The high proportion could be one of the reasons that Hyakumakidani leached high concentrations of nitrate into streams.

### ACKNOWLEDGEMENTS

We are grateful for the cooperation of the Toyama City Office and M. Takahashi in the investigation of the Hyakumakidani watershed and of the Sannokuma watershed. We also thank S. Takahara for drawing the topographic map and the geologic map.

| | | |
|---|---|---|
| Received: | July | 16, 2001 |
| Revised: | October | 18, 2001 |
| Accepted: | November | 7, 2001 |
| Published: | November | 21, 2001 |
